# Study on the plant and fish production in the aquaponic system as affected by different hydraulic loading rates

**DOI:** 10.1038/s41598-023-44707-1

**Published:** 2023-10-16

**Authors:** El-Sayed Khater, Adel Bahnasawy, Samir Ali, Wael Abbas, Osama Morsy, Amr Sabahy

**Affiliations:** 1 Agricultural and Biosystems Engineering Department, Faculty of Agriculture, Benha University, P.O. Box 13736, Moshtohor, Toukh, Kalubia Egypt; 2 Basic and Applied Science Department, College of Engineering and Technology, Arab Academy for Science and Technology and Maritime Transport (AASTMT), P.O. Box 2033, Cairo, Egypt; 3 Institute of Agricultural Engineering Research, Agriculture Research Center, Doki, Giza, Egypt

**Keywords:** Ecology, Environmental sciences, Engineering

## Abstract

Aquaponics is the combined culture of fish and plants in recirculating aquaculture systems, considered to be an innovative, eco-friendly and sustainable technology. The effect of the hydraulic loading rate (HLR) on the performance of fish and plants in the aquapoinc system was the main aim of this study. Four hydraulic loading rates were applied, 1.2, 1.8, 2.4 and 3.0 m day^−1^ under stocking density tilapia fish of 5 kg m^−3^ and lettuce population of 25 plant m^−2^ for a period of January to March, 2023. Water parameters, plant and fish parameters were determined. The most important results revealed that the highest plant nutrients removal was at HLR of 2.4 m day^−1^. The highest value of water parameters were found at the HLR of 2.4 m day^−1^. Root length increased with increasing HLR. Fresh and dry shoot and root weight values were higher at 2.4 m day^−1^ compared to other treatments under study. Meanwhile, fish growth parameter showed higher values at the HLR of 3.0 m day^−1^ compared to other treatments. The highest values of weight gain, feed growth rate, specific growth rate and feed conversion ratio were 81.72 g, 1.36 g day^−1^, 1.88% day^−1^ and 1.20 g feed g^−1^ fish, respectively, for all treatments under study.

## Introduction

Aquaponic system is an integrated fish and plant system. The water is used to rare fish is useful for plant growth. Plants serve as biofilter which converts the harmful to harmless. Nutrients which are removed by plants improve the water quality and enhance the fish production. The amount of nitrate produced in fish culture is affected by fish density, protein content in feed and fish species^1^. Aquaponics is the integration between the hydroponic cropping systems and the recirculating aquaculture systems. In this system, the dissolved nutrients in fish tank are used to supply crops with nutrients. The potential benefits of integrated production include increased revenue from the combination of fish and plant sales, minimized reliance on synthetically derived fertilizers, and both location independent and season-independent protein and vegetable production in food deserts^2,3^.

Aquaponics is a system that has advantages compared to both aquaculture and hydroponics for many reasons. It reduces the use of chemical fertilizers, decreases the agricultural run-off possibility and improve the water properties by using biofelters^4^. In this system, the fish excreta release the nutrients and the organic wastes which are broken down by micro-organisms, which are used by plants in aquaponic systems^5^. One of the system advantages; that the plants play as biofelters in separate waste solids^6^. Therefore, aquaponic system is considered an economical system, where plants and aquatic species benefit from each other, which benefit the producers. One of the best features of aquaponic is saving water and prolongs the useful life of water which reduces pollution^4,7^.

Hydraulic loading rate (HLR) is very important to achieve optimum growth of both plant and fish by improving the dissolved oxygen levels which satisfy the oxygen requirements of both fish and plants^8^.

The water discharged from fish tank contains dissolved nutrient such as nitrogen (N) and phosphorus (P), specific organic and inorganic matters, and some suspended solids (SS). These materials sources are from the uneaten feed and metabolic wastes from the fish^9,10^. These wastes accumulated in the fish tank creating negative impact on the survival and growth of fish^11^. Some nitrogen and ammonia (NH_3_) in water are the most dangerous for fish^12,13^. NH_3_ causes reduction in fish growth due to decreasing appetite and feed intake level^14^. Total ammonia nitrogen (TAN) in water is present in two forms; non-ionized ammonia (NH_3_) and ionized ammonia (NH_4_^+^)^15–17^.

In aquaponic systems, hydraulic loading rate (HLR) is a very critical factor must be optimized for optimum production because it affects the system performance^18^. Low hydraulic loading rate leads to oxygen deficiency and cause denitrification which high hydraulic loading rate reduces contact time between roots and water^19–22^. Thus, effective hydraulic loading rate could be used to achieve optimum growth of both fish and plants^23^. The oxygen concentration at inlet and outlet is very important and should be varied according to the ratio between plant and fish^5^. Reduction rates of different nutrients result in excess concentration of nutrients to the plants and accumulation of excess nutrient in fish causing fish toxic effects^24^. Thereby, adding appropriate amounts of nutrients to plant and minimizing nutrient toxicity to fish should be maintained by optimum hydraulic loading rate^20,25^.

The type of plant used in aquaponic system is very important. Lettuce is widely used because it can be harvested in short time and has fewer problems and low–medium nutrient requirements^26^. Romaine lettuce is a vegetable that is suitable for such systems^27^.

Water is very important to satisfy the fish and plant requirements from oxygen and nutrients. Effective hydraulic loading rate (HLR) is very important to achieve optimal growth for both fish and plants in the integrated aquaculture and hydroponics systems, therefore, the main aim of this work is to determine the optimum hydraulic loading rate and its relationship with plant and fish performance in the aquaponic system.

## Materials and methods

The main experiment was carried out in a greenhouse at Fish Farms and Protected Houses Center, Faculty of Agriculture Moshtohor, Benha University, Egypt (latitude 30° 21ʹ N and 31° 13ʹ E). During the period of January to March, 2023 season under the regulations of International, National and Benha University which are consistent with the national and international guidelines and legislation.

### Materials

#### System description

**Figure 1:**
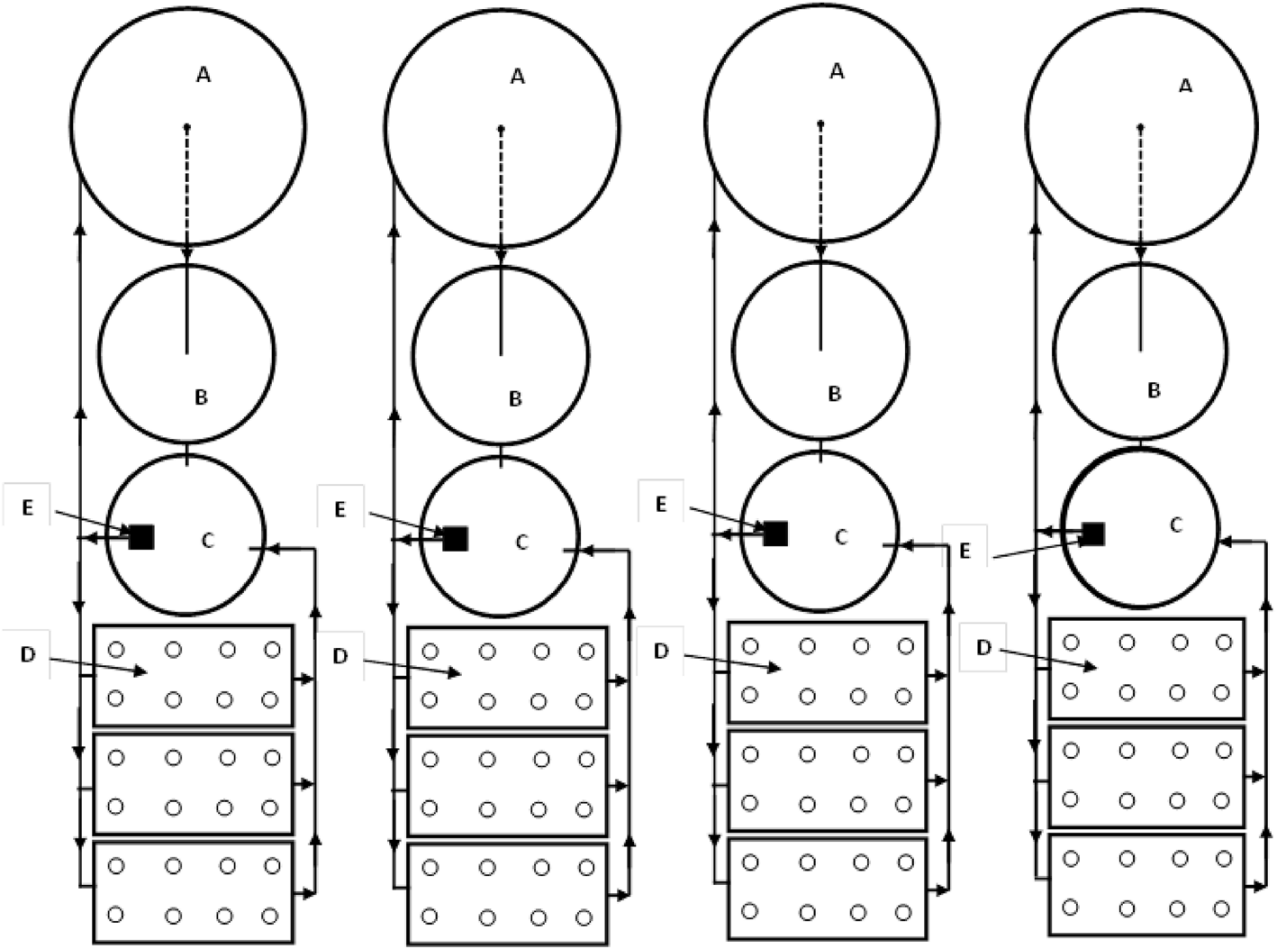
The experimental setup. (**A**) Fish tanks, (**B**) bio-sump tank, (**C**) water holding tank, (**D**) hydroponic units and (**E**) pump.

The recirculating aquaponic system which consists of fiberglass fish tanks, bio-sump tank, hydroponic units, air blower, pumps, water holding tank and reservoir, pipelines made of polyvinyl chloride were installed to connect the culture tank and hydroponic trough to recirculate the water (Fig. 1).

The system consists of four circular fiberglass tanks for fish culture, with dimensions of each tank are 1.0 m diameter and 1.0 m height. The water volume in each tank was 0.60 m^3^. There are other four circular fiberglass tanks (Bio-sump) were used in this system for solid wastes removal with dimensions of each tank are 0.75 m height and 0.70 m diameter. The water volume used in each tank was 0.25 m^3^. Polyethylene sheets were used as a media for solids removal and carry bacteria (Nitrosomonas and Nitrobacter) in the system to improve the water quality.

Figure 1 shows the hydroponic units that used for experiment which include twelve tanks made of polyethylene and rectangular in shape (dimensions of 80 cm long, 40 cm wide and 30 cm high). These tanks were placed on 1 m height from the ground with 2% slope and covered with foam slab to fix the seedlings in it. Irrigation water and solution coming from fish tank were supplied to plants from water tank with the proper nutrient solution by 0.5 hp pump (Model First QB60—Flow Rate 30 L min^−1^, Head 25 m, Power 0.5 hp, China).

The system consists of four circular polyethylene tanks of the water and nutrient solution system 500 L capacity was used for collecting the drained solution by gravity from the ends of the three systems. The air was pumped to the fish tanks and hydroponic units by using an air blower (Model C.C.P. Parma—Flow Rate 10 m^3^ h^−1^, Head 2.7 bar, Power 1.0 kW, Italy), under various pressures through air stones.

The nutrient solutions contain the required quantities for plants which include: KNO_3_, 101 g L^−1^, Ca(NO_3_)_2_, 236 g L^−1^, KH_2_PO_4_, 136 g L^−1^, K_2_SO_4_, 115 g L^−1^, MgSO_4_ 246 g L^−1^ and chelates for trace elements into preacidified groundwater (from the following ppm concentration are achieved in this formulation: N = 210, P = 31, K = 234, Ca = 200, Mg = 48, S = 64, Mn = 0.5, Fe = 14, Cu = 0.02, Zn = 0.05, B = 0.5 and Mo = 0.01). pH and EC were further adjusted to 6.0–6.5 and 800—840 ppm, respectively, after salt addition.

#### Plant and fish species

##### Lettuce plants

Lettuce (Lactuca sativa var. crispa) seedlings were gown in the plastic cups (7 cm diameter and 7 cm height) filled with peat moss under the regulations of International, National and Benha University which are consistent with the national and international guidelines and legislation. The cups were irrigated daily using water with nutrient solution. Two weeks old lettuce seedlings were planted at 25.0 plant m^−2^ in the experimental tanks^28^.

##### Nile Tilapia fish

Tilapia nilotica fingerlings (75 fingerlings for each tank with an individual weight of 40 g), which were used in the beginning of experiment. The fish was weighed every ten days and the flow rate was adjusted according to the growth rate. The daily feed rates at different fish sizes were applied according to Ref.^29^ by weighing fish and control the flow rate by valves and the feed pellet diameter was prepared according to Ref.^30^. Feeding was stopped during weighing process.

### Methods

#### Treatments

In this study, four hydraulic rates are 1.2, 1.8, 2.4 and 3.0 m day^−1^. The experimental design was a split plot with three replicates.

#### Physiochemical parameters analysis

##### Water parameters

Water temperature, pH, EC and dissolved oxygen were each day. Samples from the influent and effluent from the system were taken each ten days to measure total nitrogen (Ammonia (NH_3_), Nitrite (NO_2_) and Nitrate (NO_3_-N)), Potassium (K), Phosphorus (P), Magnesium (Mg) and Calcium (Ca) at 10 am. Water temperature and EC measured by EC Meter (Model ORION 230A—Range 0.0, 19.99 ± 0.05, USA). Water pH measured by pH Meter (Model ORION 105—Range 0.0, 9999.9 ppm ± 0.5 ppm, USA). Dissolved oxygen was recorded using a DO Meter (Model HANNA HI5421; Range: 0–90 mg L^−1^ ± 1.5%, Italy). Ammonia (NH_3_), Nitrite (NO_2_) and Phosphorus (P) measured by a Spekol 11 (Model SPEKOL 11—Range 0. 1—1000 concentration ± 1 nm λ, UK). Nitrate (NO_3_-N) content was measured by using salicylic acid as described by Ref.^31^. Potassium (K), Calcium (Ca) and Magnesium (Mg) measured by flame photometer (Model Jenway PFP7—Range 0. 1—999.9 ppm ± 0.2 ppm, USA).

##### Nutrients removal

To determine the nutrients removal rates, the following Eq. (1) was used as follows according to^32^:

1 $$ C_{Nc} = \frac{{Nc_{in} - Nc_{out} }}{n} \times Q \times 24 $$

where C_Nc_ is the nutrients removal rate, mg day^−1^ plant^−1^, Nc_in_ is the nutrients at inlet of the hydroponic unit, mg L^−1^, Nc_out_ is the nutrients at outlet of the hydroponic unit, mg L^−1^, Q is the discharge, L h^−1^, n is the number of plants

##### Plant samples

Root length was measured every ten days. To study the behaviour of root growth, their mass production and assess to which extent their roots could be grown in the growing solution.

The fresh and dry weight of shoot and root were measured at the end of the experiment. Dry weight the plants were measured by using oven dryer at 65 °C until constant weight was reached.

##### Biological factors of fish

Fish sample were taken every ten days to determine the biological parameters which include: weight gain, fish growth rate, specific growth rate, feed conversion ratio and feed efficiency ratio using the following equations:

2 $$ WG = W_{f} - W_{i} $$

3 $$ FGR = \frac{WG}{t} $$

4 $$ SGR = \frac{{\ln W_{f} - \ln W_{i} }}{t} \times 100 $$

5 $$ FCR = \frac{FI}{{WG \cdot n_{t} }} $$

6 $$ FER = \frac{{WG \cdot n_{t} }}{FI} $$

where WG is the mass gained, g, W_f_ is the mean final fish mass, g, W_i_ is the mean initial fish mass, g, FGR is the fish growth rate, g day^−1^, SGR is the specific growth rate, (% or g day^−1^), t is the time, day, FCR is the feed conversion ratio, g feed g^−1^ fish, FER is the feed Efficiency ratio, g fish g^−1^ feed, FI is the feed intake, g, n_t_ is the final number of fish in the tank.

All experimental protocols were approved by Benha university research committee and all methods used in this study were carried out according to the International, National and Benha University guidelines regulations. This study was carried out in compliance with the ARRIVE guidelines. This work is approved by the ethic committee at Benha University.

### Statistical analysis

The data were subjected to analysis using statistical package SPSS version 21 in which one way ANOVA and Duncan Multiple Range Test (DMRT) were performed at significance level of (p < 0.05) at 95% confidence limit to know the significant differences between the treatment means for different parameters according to Ref.^33^.

## Results and discussion

### Water quality parameters

In aquaponic system, water quality is one of the most important factors to determine suitable hydraulic loading rate (1.2, 1.8, 2.4 and 3.0 m day^−1^). Different water quality parameters in different treatments during the experimental period are shown in Table 1. Water temperature did not show any significant variation throughout the experimental period for the values of culture systems. It varied within a narrow range (23.41 ± 0.65 to 23.63 ± 0.81 °C). It means the variation of hydraulic loading rate did not affect the water temperature. Water temperature is one of the important factors responsible for optimum fish and plant growth. The water electrical conductivity (EC) during the experimental period varied within a range of 675.91 ± 29.16 to 695.37 ± 32.18 mg L^−1^. The water pH during the study period varied within a range of 6.51 ± 0.32 to 6.82 ± 0.48, with no marked variation among the treatments at time of sampling. Different culture systems did not show any significant effect on water pH which was found in desirable limits for fish as well as plant growth. These results agreed with those obtained by Ref.^34^. Dissolved oxygen (DO) in water during the growth period varied within a range of 6.33 ± 0.89 to 6.93 ± 0.80 mg L^−1^. Dissolved oxygen (DO) in water increases with increasing hydraulic loading rate during experimental period. It could be seen that the DO in water was increased from 6.33 ± 0.89 to 6.93 ± 0.80 mg L^−1^, when the hydraulic loading rate increased from 1.2 to 3.0 m day^−1^, respectively. Dissolved oxygen did not show any significant variation throughout the growth period for the values of culture systems. DO varied within narrow range and they were found within optimum limits for the fish as well as plant culture.

**Table 1:** Water quality parameters.

Parameter	Hydraulic loading rate, m day^−1^			
	1.2	1.8	2.4	3.0
Temperature, °C	23.63 ± 0.81^a^	23.57 ± 0.76^a^	23.41 ± 0.65^a^	23.40 ± 0.44^a^
EC, mg L^−1^	695.37 ± 32.18^a^	690.19 ± 25.65^a^	675.91 ± 29.16^a^	691.22 ± 35.07^a^
pH	6.51 ± 0.32^a^	6.54 ± 0.29^a^	6.76 ± 0.51^a^	6.82 ± 0.48^a^
DO, mg L^−1^	6.33 ± 0.89^a^	6.58 ± 0.76^a^	6.71 ± 0.96^a^	6.93 ± 0.80^a^
NH_3_, mg L^−1^	0.026 ± 0.001^b^	0.025 ± 0.001^a^	0.023 ± 0.001^a^	0.024 ± 0.002^a^
Nitrite, mg L^−1^	0.41 ± 0.06^b^	0.29 ± 0.04^a^	0.29 ± 0.05^a^	0.31 ± 0.07^a^
Nitrate, mg L^−1^	16.33 ± 2.59^b^	16.10 ± 3.81^b^	14.48 ± 2.61^a^	16.11 ± 4.84^b^
Phosphorus, mg L^−1^	8.92. ± 3.31^a^	10.77 ± 2.85^b^	11.02 ± 1.26^b^	10.73 ± 1.99^b^
Potassium, mg L^−1^	47.25 ± 4.02^d^	45.90 ± 4.87^c^	39.81 ± 4.55^a^	43.61 ± 5.02^b^
Calcium, mg L^−1^	32.61 ± 5.30^a^	34.09 ± 5.91^a^	40.11 ± 2.38^b^	40.78 ± 3.83^b^
Magnesium, mg L^−1^	21.58 ± 4.61^a^	24.52 ± 3.64^c^	24.99 ± 3.07^c^	23.74 ± 3.17^b^

Means on the same row with different superscripts are significantly different (p < 0.05).

Ammonia-N generation rate in the system was higher for 1.2 m day^−1^ hydraulic loading rate (T1) than other treatments. Ammonia-N generation rate depends on the feeding rate in the system which was adjusted after each sampling as per the fish body weight. Significantly lowest Nitrite-N concentration was observed for 1.2 m day^−1^ hydraulic loading rate (0.41 ± 0.06 mg L^−1^) as compared to other treatments (1.8, 2.4 and 3.0 m day^−1^ hydraulic loading rate). Nitrate–N concentration for 1.2, 1.8 and 3.0 m day^−1^ hydraulic loading rates were significantly higher than 1.8 m day^−1^ hydraulic loading rate. Nitrate–N is relatively less toxic to fish and is not a health hazard except at exceedingly high levels above 300 mg L^−1^^35,36^. The phosphorus concentration values were 8.92 ± 3.31,10.77 ± 2.85, 11.02 ± 1.26 and 10.73 ± 1.99 mg L^−1^ for 1.2, 1.8, 2.4 and 3.0 m day^−1^ hydraulic loading rates, respectively. Phosphorus concentration was found significantly higher in the 1.8, 2.4 and 3.0 m day^−1^ hydraulic loading rates as compared to 1.2 m day^−1^ hydraulic loading rates. Potassium concentration values were 47.25 ± 4.02, 45.90 ± 4.87, 39.81 ± 4.55 and 43.61 ± 5.02 mg L^−1^ for 1.2, 1.8, 2.4 and 3.0 m day^−1^ hydraulic loading rates, respectively. The highest values of calcium and magnesium concentration (40.78 ± 3.83 and 24.99 ± 3.07 mg L^−1^) were found with 2.4 m day^−1^ hydraulic loading rate. These results agreed with those obtained by Ref.^37^.

### Nutrients removal rate

Table 2 shows the nitrogen (N), phosphorus (P), potassium (K), calcium (Ca) and magnesium (Mg) removal rate by lettuce plants for different hydraulic loading rate (1.2, 1.8, 2.4 and 3.0 m day^−1^) during the growth period. The results indicate that the nitrogen (N) removal rate by lettuce plants values were 776.73 ± 31.60, 813.26 ± 20.49, 862.57 ± 29.05 and 821.70 ± 22.77 mg plant^−1^ for 1.2, 1.6, 2.4 and 3.0 m day^−1^ hydraulic loading rate, respectively, during growth period.

**Table 2:** The nutrients consumption rate of lettuce plants grown in different hydraulic loading rates.

Parameter	Hydraulic loading rate, m day^−1^			
	1.2	1.8	2.4	3.0
Nutrients consumption rate, mg plant^−1^				
Nitrogen	776.73 ± 31.60	813.26 ± 20.49	862.57 ± 29.05	821.70 ± 22.77
Phosphorus	723.26 ± 22.14	752.45 ± 28.81	781.26 ± 20.33	764.92 ± 23.41
Potassium	2429.38 ± 77.00	2621.14 ± 61.37	2732.87 ± 71.56	2632.79 ± 46.27
Calcium	527.90 ± 19.82	565.99 ± 26.90	576.91 ± 25.52	574.14 ± 21.03
Magnesium	420.77 ± 12.99	459.37 ± 21.03	456.60 ± 17.58	463.22 ± 19.32

Means on the same row with different superscripts are significantly different (p < 0.05).

The results indicate that the phosphorus (P) removal rate by lettuce plants values were 723.26 ± 22.14, 752.45 ± 28.81, 781.26 ± 20.33 and 764.92 ± 23.41 mg plant^−1^ for 1.2, 1.6, 2.4 and 3.0 m day^−1^ hydraulic loading rate, respectively, during growth period. The potassium (K) removal rate by lettuce plants values were 2429.38 ± 77.00, 2621.14 ± 61.37, 2732.87 ± 71.56 and 2632.79 ± 46.27 mg plant^−1^ for 1.2, 1.6, 2.4 and 3.0 m day^−1^ hydraulic loading rate, respectively, during growth period. The calcium (Ca) removal rate by lettuce plants values were 527.90 ± 19.82, 565.99 ± 26.90, 576.91 ± 25.53 and 574.14 ± 21.03 mg plant^−1^ for 1.2, 1.6, 2.4 and 3.0 m day^−1^ hydraulic loading rate, respectively, during growth period. The magnesium (Mg) consumption rate by lettuce plants values were 420.77 ± 12.99, 429.37 ± 21.03, 466.60 ± 17.58 and 463.22 ± 19.32 mg plant^−1^ for 1.2, 1.6, 2.4 and 3.0 m day^−1^ hydraulic loading rate, respectively, during growth period.

The results indicate that, the N, P, k, Ca and Mg removal rate increases with increasing hydraulic loading rate until it reached the peak with 2.4 m day^−1^ hydraulic loading rate and then decreased during growth period. The trend of these results agreed with those obtained by Ref.^24^. The highest values of N, P, k, Ca and Mg removal rate were 862.57 ± 29.05, 781.26 ± 20.33, 2732.87 ± 71.56, 576.91 ± 25.52 and 466.60 ± 17.58 mg plant^−1^ were found with 2.4 m day^−1^ of hydraulic loading rate, while, the lowest values of N, P, k, Ca and Mg removal rate were 776.73 ± 31.60, 723.26 ± 22.14, 2429.38 ± 77.00, 527.90 ± 19.82 and 420.77 ± 12.99 mg plant^−1^ were found with 1.2 m day^−1^ of hydraulic loading rate. These results agreed with those obtained by Ref.^38^ compared three flow rates (1.0, 1.5 and 2.0 L h^−1^ plant^−1^) and found fish as well as lettuce growth increased with increasing water flow rate from 1.0 to 1.5 L h^−1^ plant^−1^. Lower nutrients consumption rate of lettuce plants at lower hydraulic loading rate (1.2 m day^−1^) may be because of nutrient solution stay a long time under roots, the nutrient solution insufficient to supply the plant by nutrients. Similar results were also found by Refs.^38,39^, with plant registered lower amount of nutrients consumption rates in lower hydraulic loading rate treatment.

### Plant growth parameters

#### Root length

**Figure 2:**
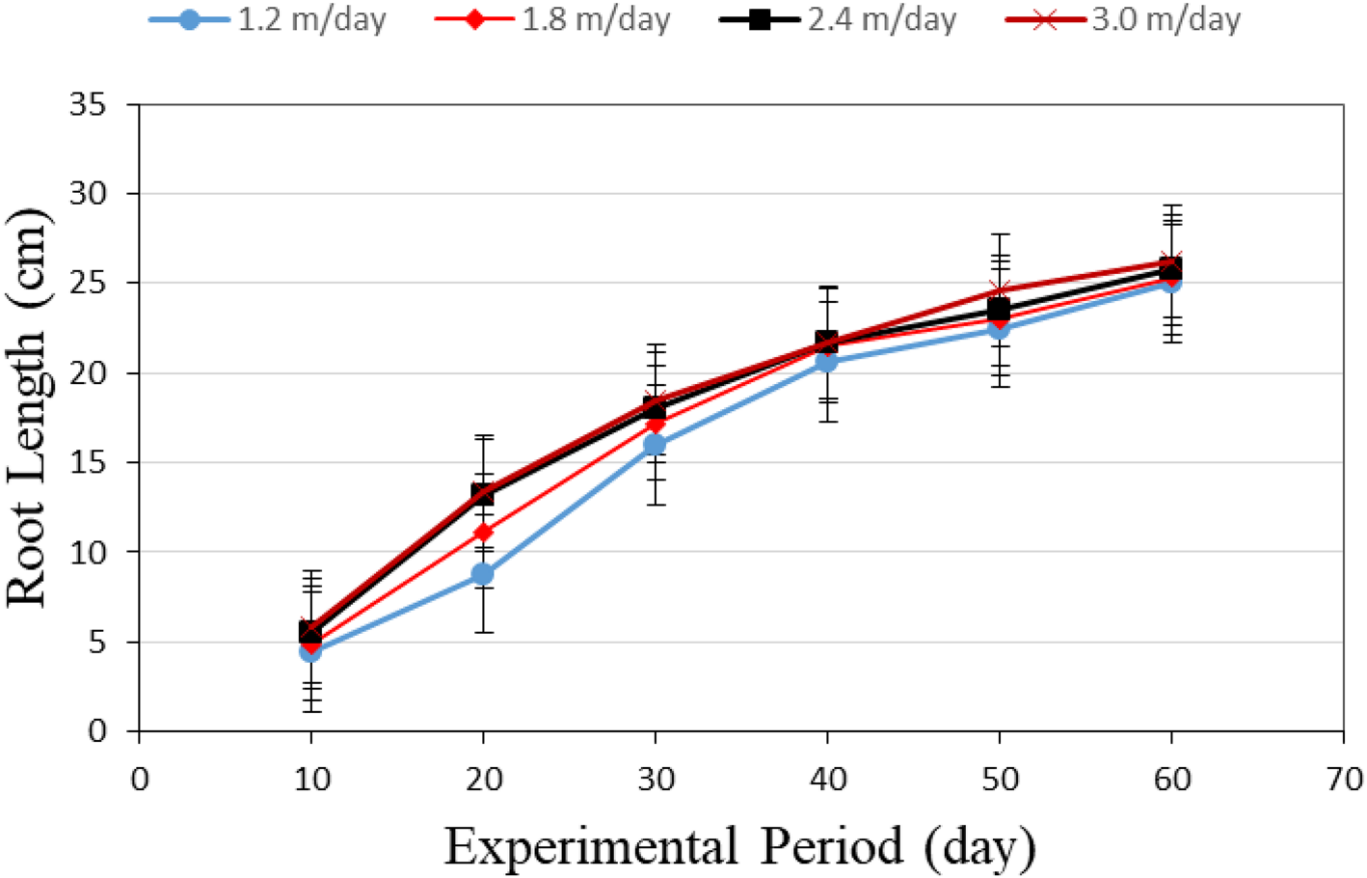
The root length of Lettuce plants grown in different hydraulic loading rate.

Figure 2 shows the root length of lettuce plants grown in different hydraulic loading rate (1.2, 1.8, 2.4 and 3.0 m day^−1^) during the growth period. The results indicate that the root of the lettuce plant grown in different hydraulic loading rate increases with increasing hydraulic loading rate and plant age. It could be seen that the root length of lettuce plants significantly increased from 4.45 ± 0.52 to 24.98 ± 2.09, 4.93 ± 0.39 to 25.31 ± 2.17 and 5.49 ± 0.45 to 25.74 ± 2.21, 5.86 ± 0.51 to 26.21 ± 2.54 cm, when the lettuce plant age increased from 10 to 60 days, respectively, at 1.2, 1.8, 2.4 and 3.0 m day^−1^ hydraulic loading rate. It was noticed that there was not any overlapping (interference) between roots of the growing plants as a result of choosing a suitable distance (25 cm) apart between plants during different growth stages. If there is any overlapping existed it was very limited (not more than 5.0%). These results were in agreement with^28^ found that the plant spacing for lettuce was (20–25 cm).

Regarding the hydraulic loading rate, the results indicate that the root length increases with increasing hydraulic loading rate. It observed that when the hydraulic loading rate increased from 1.2 to 3.0 m day^−1^, the length of root significantly increased from 4.45 ± 0.52 to 5.86 ± 0.51 and 24.98 ± 2.09 to 26.21 ± 2.54 cm after 10 and 60 days, respectively, from transplanting. Endut et al.^19^ mentioned that flow rate has a significant influence on the plant growth, and it was found that their best performance in terms of plant growth was at a flow rate of 1.6 L min^−1^. Khater et al.^32^ also observed comparable results for tomato plant growth in very low constant flow rates. The author noticed that when flow rates increased, root length of tomato plant also increased. However, in the present study, the maximum root length was obtained at 3.0 m day^−1^ hydraulic loading rate. The highest value of root length was 26.21 ± 2.54 cm was found with 3.0 m day^−1^ of hydraulic loading rate, while, the lowest value of root length was 24.98 ± 2.09 cm was found with 1.2 m day^−1^ of hydraulic loading rate. Generally, the growth of root system of the plant in a solution has optimum conditions depending on the amount of nutrients available to the roots its oxygen supply, the osmotic pressure of solution and its temperature. These results agreed with those obtained by Ref.^40^.

Multiple regression analysis was carried out to obtain a relationship between the root length of lettuce plants as dependent variable and different both of hydraulic loading rate (1.2, 1.8, 2.4 and 3.0 m day^−1^) and experimental period (1–60 day) as independent variables. The best fit for this relationship is presented in the following equation:

7 $$ R{\text{L}} = 0.78 + 0.40T + 1.21HLR\quad {\text{R}}^{2} = 0.94 $$

where RL is the root length of lettuce plant, cm; T is the lettuce plant age, day; HLR is the hydraulic loading rate, m day^−1^

#### Fresh and dry weight of shoot

**Figure 3:**
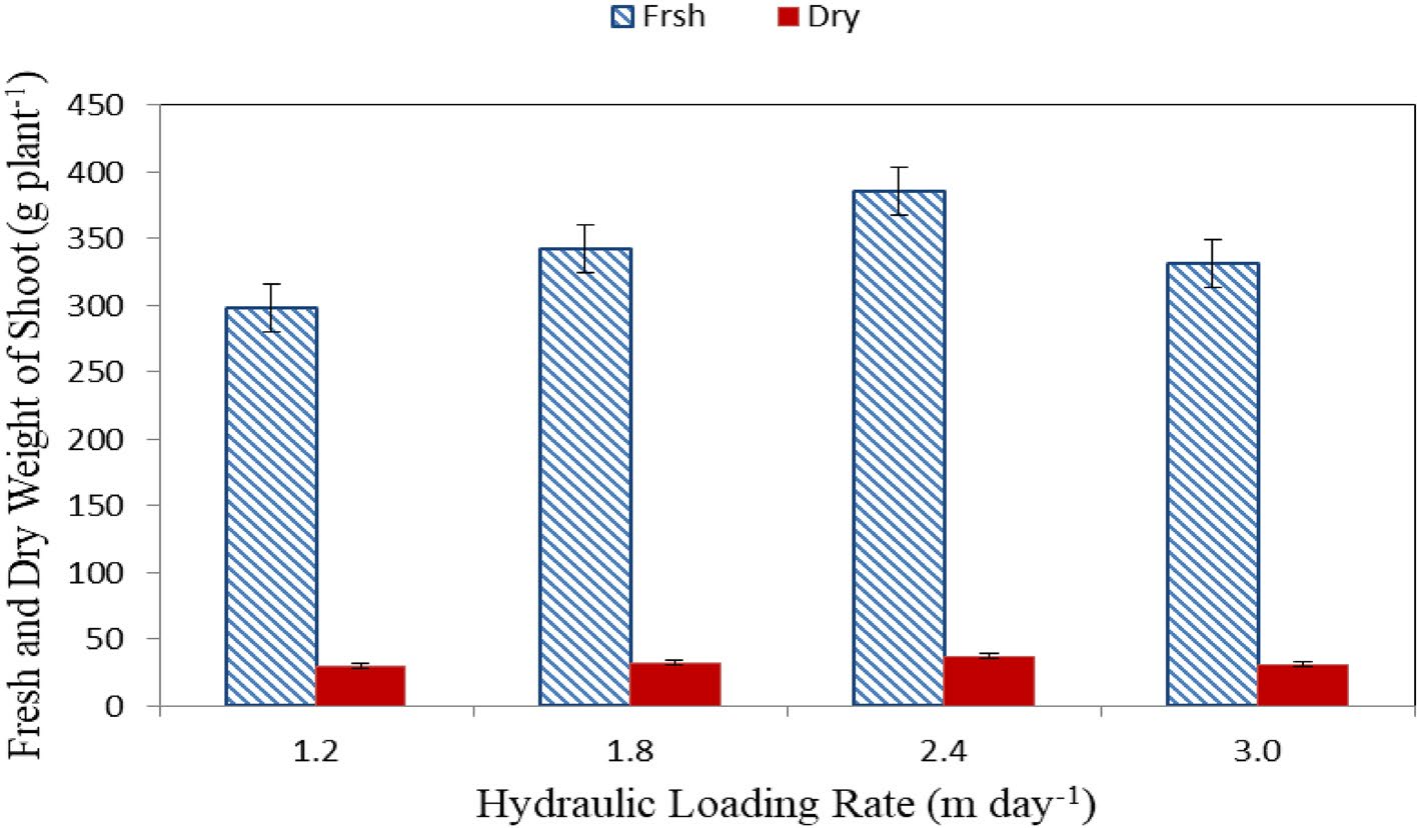
Fresh and dry weight of shoot of lettuce plants.

Figure 3 shows the fresh and dry weight of shoot of lettuce plants grown in different hydraulic loading rate (1.2, 1.8, 2.4 and 3.0 m day^−1^) at the end of growth period (60 days). The results indicate that the fresh and dry of shoot of lettuce plants grown with 2.4 m day^−1^ hydraulic loading rate (T3) were better than those of different hydraulic loading rate (1.2, 1.8 and 3.0 m day^−1^). It could be observed that the fresh weight of shoot of lettuce plants were 298.20 ± 12.94, 342.03 ± 15.71, 385.69 ± 13.05 and 331.32 ± 15.66 g plant^−1^ for 1.2, 1.8, 2.4 and 3.0 m day^−1^ hydraulic loading rate, respectively, at the end of growth period. While, the dry weight of shoot of lettuce plants were 29.56 ± 2.35, 31.88 ± 2.29, 37.05 ± 3.41 and 30.98 ± 3.28 g plant^−1^ for 1.2, 1.8, 2.4 and 3.0 m day^−1^ hydraulic loading rate, respectively, at the end of growth period. This helps explain yield and growth of lettuce plants differences from various solutions. Generally, the growth of lettuce plant in a solution has optimum conditions depending on the amount of nutrients available to the plants and their balance in addition to sufficient, oxygen supply, the appropriate osmotic pressure of solution and its temperature. These results were in agreement with Ref.^41^.

The results indicate that, the fresh and dry weight of shoot increases with increasing hydraulic loading rate until it reached the peak with 2.4 m day^−1^ hydraulic loading rate and then decreased at the end of growth period. It could be seen that, the highest values of fresh and dry weight of shoot (385.69 ± 13.05 and 37.05 ± 3.41 g plant^−1^) were found with 2.4 m day^−1^ of hydraulic loading rate, while, the lowest values of fresh and dry weight of shoot (298.20 ± 12.94 and 29.56 ± 2.35 g plant^−1^) were found with 1.2 m day^−1^ of hydraulic loading rate. These results agreed with those obtained by Refs.^38,42^ whose found that the fresh and dry weight of shoot increases with increasing hydraulic loading rate until it reached the peak.

#### Fresh and dry weight of root

**Figure 4:**
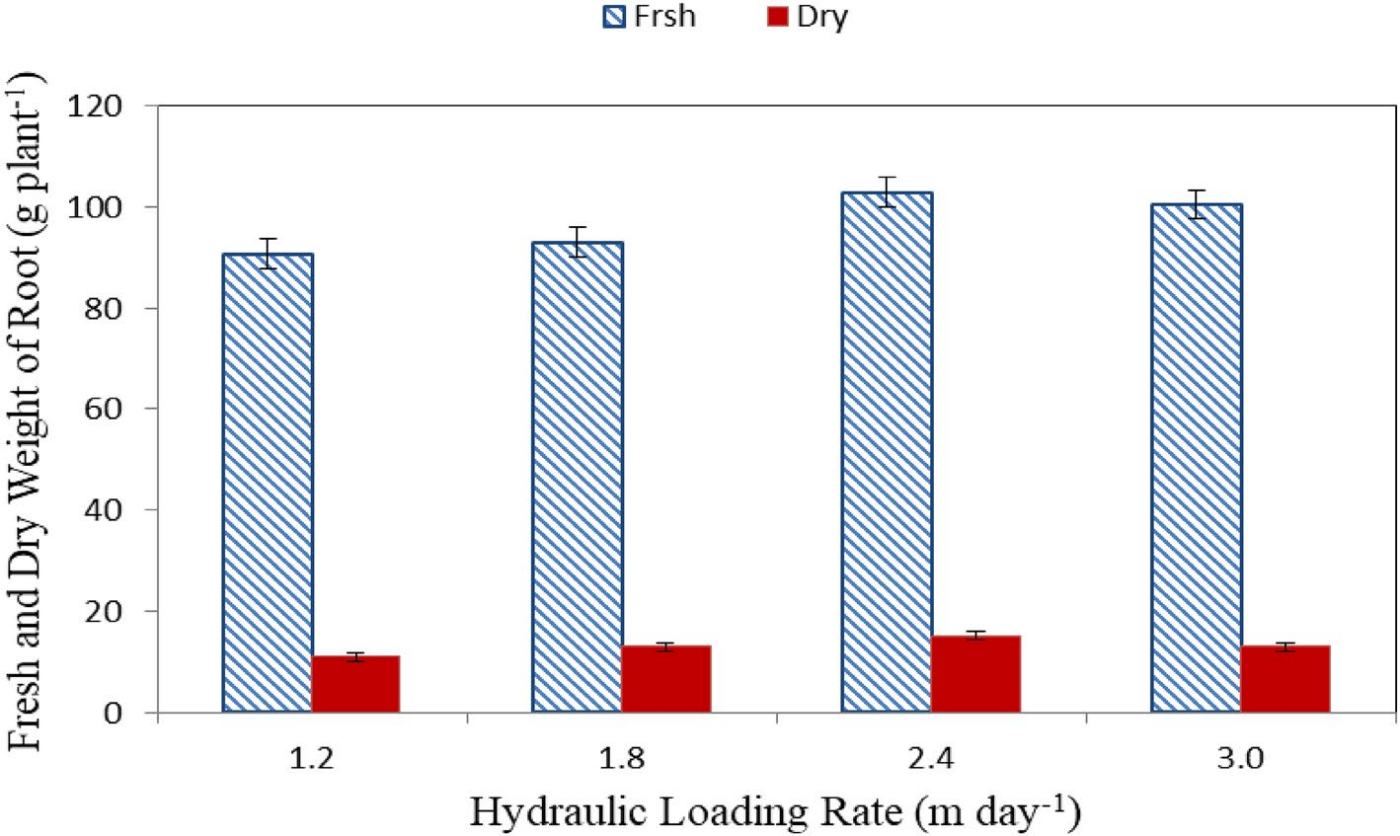
Fresh and dry weight of root of lettuce plants.

Figure 4 shows the fresh and dry weight of shoot of lettuce plants grown in different hydraulic loading rate (1.2, 1.8, 2.4 and 3.0 m day^−1^) at the end of growth period (60 days). The results indicate that the fresh and dry of shoot of lettuce plants grown with 2.4 m day^−1^ hydraulic loading rate (T3) were better than those of different hydraulic loading rate (1.2, 1.8 and 3.0 m day^−1^). It could be observed that the fresh weight of root of lettuce plants were 90.68 ± 5.02, 93.11 ± 5.5.25, 102.91 ± 3.08 and 100.44 ± 5.01 g plant^−1^ for 1.2, 1.8, 2.4 and 3.0 m day^−1^ hydraulic loading rate, respectively, at the end of growth period. While, the dry weight of root of lettuce plants were 10.97 ± 1.98, 12.99 ± 2.02, 15.06 ± 2.43 and 13.01 ± 1.94 g plant^−1^ for 1.2, 1.8, 2.4 and 3.0 m day^−1^ hydraulic loading rate, respectively, at the end of growth period.

The results indicate that, the fresh and dry weight of root increases with increasing hydraulic loading rate until it reached the peak with 2.4 m day^−1^ hydraulic loading rate and then decreased at the end of growth period. It could be seen that, the highest values of fresh and dry weight of root (102.91 ± 3.08 and 15.06 ± 2.43 g plant^−1^) were found with 2.4 m day^−1^ of hydraulic loading rate, while, the lowest values of fresh and dry weight of root (90.68 ± 5.02 and 10.97 ± 1.98 g plant^−1^) were found with 1.2 m day^−1^ of hydraulic loading rate. These results agreed with those obtained by Refs.^38,43^ whose found that the fresh and dry weight of root increases with increasing hydraulic loading rate until it reached the peak.

### Fish growth parameters

Table 3 shows the total weight gain, fish growth rate, specific growth rate, feed conversion ratio and feed efficiency ratio for different hydraulic loading rate (1.2, 1.8, 2.4 and 3.0 m day^−1^) at the end growing period. The results indicate that the total weight gain of fish with different hydraulic loading rate increases with increasing hydraulic loading rate. It could be seen that the total weight gain of fish significantly increased from 63.01 ± 2.06 to 81.72 ± 2.57 g, when the hydraulic loading rate increased from 1.2 to 3.0 m day^−1^, respectively, at the end of experimental period.

**Table 3:** Fish growth parameters.

Parameter	Hydraulic loading rate, m day^−1^			
	1.2	1.8	2.4	3.0
Initial weight, g	40.00 ± 1.27^a^	40.00 ± 1.27^a^	40.00 ± 1.27^a^	40.00 ± 1.27^a^
Final weight, g	103.01 ± 2.11^a^	110.38 ± 1.84^b^	119.16 ± 1.73^c^	121.72 ± 2.44^c^
Total weight gain, g	63.01 ± 2.06^a^	70.38 ± 2.11^b^	79.16 ± 1.98^c^	81.72 ± 2.57^d^
FGR, g day^−1^	1.05 ± 0.32^a^	1.17 ± 0.31^b^	1.32 ± 0.44^c^	1.36 ± 0.29^c^
SGR, % day^−1^	1.58 ± 0.25^a^	1.69 ± 0.34^b^	1.82 ± 0.19^c^	1.85 ± 0.21^c^
FCR, g feed g^−1^ fish	0.92 ± 0.11^a^	1.03 ± 0.09^b^	1.16 ± 0.13^c^	1.20 ± 0.12^c^
FER, g fish g^−1^ feed	1.09 ± 0.07^c^	0.97 ± 0.06^b^	0.86 ± 0.08^a^	0.83 ± 0.08^a^

Means on the same raw with different superscripts are significantly different (p < 0.05).

The results indicate that the fish growth rate (FGR) were 1.05 ± 0.32, 1.17 ± 0.31, 1.32 ± 0.44 and 1.36 ± 0.29 g day^−1^ for 1.2, 1.8, 2.4 and 3.0 m day^−1^ hydraulic loading rate, respectively, at the end of growth period. The expected FCR for tilapia ranges from 1.0 to 2.0 g day^−1^^44^. The specific growth rate (SGR) was 1.58 ± 0.25, 1.69 ± 0.34, 1.82 ± 0.19 and 1.85 ± 0.21% day^−1^ for 1.2, 1.8, 2.4 and 3.0 m day^−1^ hydraulic loading rate, respectively, at the end of growth period. These values are in agreement with obtained by Refs.^1,45^**,** which ranged from 1.68 to 1.80% day^−1^. The feed conversion ratio (FCR) was 0.92 ± 0.11, 1.03 ± 0.09, 1.16 ± 0.13 and 1.20 ± 0.12 g feed g^−1^ fish for 1.2, 1.8, 2.4 and 3.0 m day^−1^ hydraulic loading rate, respectively, at the end of growth period. These values are in agreement with obtained by Ref.^46^**,** whose found the FCR ranged from 1.01 to 1.02 g feed g^−1^ fish. The feed efficiency ratio (FER) was 1.09 ± 0.07, 0.97 ± 0.06, 0.86 ± 0.08 and 0.83 ± 0.08 g fish g^−1^ feed for 1.2, 1.8, 2.4 and 3.0 m day^−1^ hydraulic loading rate, respectively, at the end of growth period. Better fish growth performance with 3.0 m day^−1^ hydraulic loading rate may be as results of better water quality parameters. These results were in agreement with Ref.^25^.

From statistical analysis, there were no significant different between 2.4 and 3.0 m day^−1^ hydraulic loading rate on the fish growth rate, specific growth rate, feed conversion ratio and feed efficiency ratio, meanwhile, there were significant differences between 1.2, 1.8 and 2.4 m day^−1^ hydraulic loading rate on the fish growth rate, specific growth rate, feed conversion ratio and feed efficiency ratio.

## Conclusions

This work was done successively to find out the relationship between hydraulic loading rate and the aquaponic performance in terms of fish and plant growth. It is concluded that using HLR of 2.4 m day^−1^ gave the best results in both fish and plants growth, where it recorded higher nutrients removal rates and higher specific growth rate (SGR) compared to other hydraulic loading rate levels under this study. It is recommended to pay more attention to the effect of hydraulic loading rate levels and their effects on the aquaponic systems. Considering plant and fish growth parameters as well as nutrients removal percentages, a hydraulic loading rate of 2.4 m day^−1^ was found to be the optimum in aquaponics.

## Data Availability

The datasets used and/or analyzed during the current study available from the corresponding author on reasonable request.
